# Human placental mesenchymal stem cells ameliorate chemotherapy-induced damage in the testis by reducing apoptosis/oxidative stress and promoting autophagy

**DOI:** 10.1186/s13287-021-02275-z

**Published:** 2021-03-20

**Authors:** Jiafeng Lu, Zhenxing Liu, Mingkai Shu, Liya Zhang, Wenjuan Xia, Liuna Tang, Jincheng Li, Boxian Huang, Hong Li

**Affiliations:** 1 Center of Reproduction and Genetics, The affiliated Suzhou Hospital of Nanjing Medical University, Suzhou Municipal Hospital, Gusu School, State Key Laboratory of Reproductive Medicine, Nanjing Medical University, Suzhou, 215002 China; 2 Medical College of Soochow University, 199 Renai Road, Industrial Park District, Suzhou, 215123 China

**Keywords:** Busulfan, Spermatogenesis, Human placental mesenchymal stem cells, Autophagy, Reactive oxygen species, Apoptosis

## Abstract

**Background:**

The side effects of busulfan on male reproduction are serious, so fertility preservation in children undergoing busulfan treatment is a major worldwide concern. Human placental mesenchymal stem cells (hPMSCs) have advantages such as stable proliferation and lower immunogenicity that make them an ideal material for stimulating tissue repair, especially restoring spermatogenesis. The protective effects of hPMSCs in busulfan-induced Sertoli cells and in busulfan-treated mouse testes have not been determined. Our study aimed to elaborate the protective effect and potential mechanisms of hPMSCs in busulfan-treated testes and Sertoli cells.

**Methods:**

First, we developed a mouse model of busulfan-induced testicular toxicity in vivo and a mouse Sertoli cell line treated with busulfan in vitro to assess the protective effect and mechanisms of hPMSC treatment on spermatogenesis. Then, the length, width, and weight of the testes were monitored using Vernier calipers. Furthermore, at 1 week and 4 weeks after the transplantation of hPMSCs, histological sections of testes were stained with hematoxylin-eosin, and the seminiferous tubules with fluid-filled cavities were counted. Through ELISA analysis, testosterone levels and MDA, SOD, LDH, and CAT activities, which are associated with ROS, were detected. Markers of ROS, proliferation (Ki67), and apoptosis (Annexin V) were evaluated by FACS. Next, the fluorescence intensity of proliferation markers (BrdU and SCP3), an antioxidant marker (SIRT1), a spermatogenesis marker (PLZF), and autophagy-related genes (P62 and LC3AB) were detected by fluorescence microscopy. The mRNA expression of γ-H2AX, BRCA1, PARP1, PCNA, Ki67, P62, and LC3 was determined by qRT-PCR.

**Results:**

hPMSCs restored disrupted spermatogenesis, promoted improved semen parameters, and increased testosterone levels, testis size, and autophagy in the testis toxicity mouse model induced by busulfan. hPMSCs suppressed the apoptosis of Sertoli cells and enhanced their rate of proliferation in vitro. Additionally, hPMSCs protected against oxidative stress and decreased oxidative damage in the testis toxicity mouse model induced by busulfan. Furthermore, hPMSCs increased the expression of proliferation genes (PCNA and KI67) and decreased the mRNA levels of apoptotic genes such as γ-H2AX, BRCA1, and PARP1.

**Conclusions:**

This research showed that hPMSC injection ameliorated busulfan-induced damage in the testis by reducing apoptosis/oxidative stress and promoting autophagy. The present study offers an idea for a new method for clinical treatment of chemotherapy-induced spermatogenesis.

**Supplementary Information:**

The online version contains supplementary material available at 10.1186/s13287-021-02275-z.

## Background

Spermatogenesis of mammals is a profoundly organized and dynamic cell differentiation process with three stages: mitosis, meiosis, and spermatogenesis [1]. In the process of spermatogenesis, Sertoli cells play crucial roles that include regulating multidirectional differentiation and spontaneous recovery of spermatogonia stem cells (SSCs), providing nourishment as well as fundamental support for developing germ cells and so on [2]. Deviation in these processes could lead to abnormal characteristics in the morphology, function, and motility of the sperm [3]. For instance, the spermatogenesis process is susceptible to toxicity, contamination, anticancer medicine, and other factors [4–6]. In particular, spermatogonial progenitor cells (SPCs) are sensitive to the toxicity of busulfan, but the molecular mechanism remains largely unknown [7].

Busulfan is a common chemotherapeutic drug that prevents cellular division, and consequently, germ cells, which have high rates of division, are vulnerable to busulfan [8]. Several studies have focused on the mechanism of busulfan toxicity, such as ROS-induced apoptosis [9]. Nevertheless, the potential mechanism induced by busulfan is very intricate since it probably involves multiple biological reactions, e.g., reactive oxygen species, proliferation, apoptosis, and autophagy [10]. Thus, the side effects of busulfan on male reproduction are so severe that fertility preservation in children undergoing busulfan treatment is a major worldwide concern.

Mesenchymal stem cells (MSCs) hold great promise for rehabilitating the microenvironment of spermatogenesis. For instance, mesenchymal stem cells derived from the human placenta are in clinical trials for a variety of restorative applications [11]. Compared with MSCs from other sources, human placental mesenchymal stem cells (hPMSCs) have the advantages of stable proliferation and low immunogenicity. These characteristics of hPMSCs make them an ideal material for stimulating tissue repair. Our group confirmed that hPMSCs ameliorate premature ovarian insufficiency via NRF2/HO-1 activation [12]. However, the mechanism by which hPMSCs restore spermatogenesis in busulfan-induced mouse testes is not clear.

Autophagy is a highly conserved membrane-trafficking process for degrading long-lived proteins and organelles by lysosomes. Autophagy starts with the development of a vesicle with a double membrane, which is named an autophagosome. The autophagosome moves along cytoskeletal structures and merges with lysosomes, creating an autolysosome [13]. To date, more than forty autophagy-related (ATG) proteins have been identified [14, 15]. Upon induction of autophagy in mammals, LC3 is activated by ATG3 and ATG7 and attaches to the lipid-containing membrane. The lipid-containing membrane serves as a scaffold to establish phagophores that become enclosed autophagosomes. Autophagy participates in many non-pathological processes, such as preimplantation development [16], ectoplasmic specialization assembly in Sertoli cells [17], and spermatid differentiation [18]. In addition, oxidative stress above normal physiological levels can damage sperm DNA, leading to male infertility [19]. A previous study also showed that apoptosis is a key physiological process in the development of testes [20]. However, the function of autophagy, apoptosis, and oxidative stress in busulfan-induced spermatogenesis remains largely unknown.

To date, the protective effects of hPMSCs in mouse testes treated with busulfan and in mouse Sertoli cells induced by busulfan have not been elaborated. Therefore, our research aimed to identify whether hPMSCs could ameliorate chemotherapy-induced damage in the testes of mice by reducing apoptosis/oxidative stress and promoting autophagy.

## Methods

### Preparation of hPMSCs

After being dissected and immediately placed in solution, samples of the human placenta were thoroughly rinsed in PBS containing antibiotic-antimycotic (100 U/ml penicillin G and 100 mg/ml streptomycin; Thermo Fisher Scientific) for 60 min on ice. The samples were split into quadrants, and then, the villous chorion and chorionic plate were diced to small pieces no more than 1 mm in length after the removal of the amniotic membrane layer. To release the cells, the minced tissues were enzymatically digested by trypsin (10 g) in culture medium for 60 min at 37 °C, and dispase (4 mg/ml, Thermo Fisher Scientific) plus Collagenase Type IV (3 mg/ml, Thermo Fisher Scientific) was added. The samples were centrifuged for 5 min at room temperature in culture medium containing 10% fetal bovine serum (FBS) before the reaction was ended. Approximately 3 × 10^7^ cells were transplanted into each 10-cm cell culture dish. The cells started growing adherently after 3 days, and within 1 week, cell clusters formed. All experiments were performed using 3rd–4th-generation hPMSCs.

### RNA extraction and reverse transcription-polymerase chain reaction (RT-PCR)

After hPMSC treatment, a Qiagen RNeasy Mini Kit (Qiagen, USA) was used to extract total RNA from the testes of the four groups of mice. A Prime-Script RT Reagent Kit (Takara, Japan) was used to reverse transcribe RNA into cDNA, SYBR Premix Ex Taq (Takara, Japan) was used to perform quantitative real-time polymerase chain reaction (PCR) utilizing a Thermal Cycler Dice Real-Time System (Takara, Japan), and the 2^-ΔΔCt^ calculation method was employed to analyze the data. The primer sequences for the genes γ-H2AX, BRCA I, PARP1, PCNA, KI67, P62, and LC3 are shown in Supplementary Table.

### Sertoli cell preparation and treatment

The TM4 mouse Sertoli cell line (Procell, China) was acquired and cultured at 1 × 10^5^ cells per well utilizing specific complete medium for TM4 (Procell, China) in an incubator. The cell medium was changed every 2 days, and the cells were trypsinized and passaged at 80–90% confluence. Busulfan was added to Sertoli cells based on a previously described approach with slight alterations [21]. Briefly, Sertoli cells were treated with 10^− 4^ μM busulfan and cultured in an incubator for 48 h. After that, hPMSCs were cocultured with the busulfan-induced Sertoli cells for 48 h using a Transwell system. The Sertoli cells were then divided into three groups: the control group (untreated), BU group (busulfan-treated), and BU/hPMSC group (treated with hPMSCs after busulfan treatment).

### Experimental mouse model

Nanjing Medical University provided male C57BL/6 mice. They were fed as described in our previous study [22]. A mouse model of testicular toxicity induced by busulfan was established using the previously described approach with some alterations. Briefly, busulfan was dissolved in DMSO and diluted with distilled water to 5 mg/mL. Busulfan (40 mg/kg) was injected into the enterocoelia of mice. Approximately 5 × 10^6^ hPMSCs suspended in normal saline were injected into the testes of mice, which were subsequently sacrificed at 1 or 4 weeks after the hPMSC treatment. Consequently, the mice were divided into three groups: the control group (untreated), BU group (busulfan treated), and BU/hPMSC group (treated with hPMSCs after busulfan treatment).

### Testes measurement and histological analysis

The blood and testes were acquired for ELISA detection and HE staining, respectively. Euthanasia was used on all mice, and the blood, testes, and epididymis were collected for follow-up experiments. Furthermore, the length, width, and weight of the testes were measured using Vernier calipers. After the transplantation of hPMSCs, the testes of mice were fixed with paraformaldehyde. The tissues were dehydrated and then clarified by xylene and paraffin-embedded, and the paraffin-embedded blocks were cut into slices of 5-μm thickness. As described in a previous study, slices of 5-μm thickness were stained with hematoxylin-eosin and examined with an optical microscope [23]. Five typical sections containing convoluted tubules with vacuoles were examined from each testis.

### ELISA analysis

Serum levels of testosterone, superoxide dismutase (SOD), malondialdehyde (MDA), catalase (CAT), and lactate dehydrogenase (LDH) were measured by ELISA (Cayman Chemical, USA). In short, 50-μl culture medium or serum was added to the plate and incubated at 37 °C for 30 min. Then, the wells were washed 5 times for 10 s each, and the cells were incubated with 50 mol/l HRP-coupling reagent at 37 °C for 60 min. The wells were washed five times for 10 s each and incubated with a mixture of substrate B and A solution (50 mol/L) at 37 °C for 30 min; last, the reaction was ended by adding 50 μl stop solution. Finally, the absorbance was measured with a spectrophotometer (BioTek, USA).

### Fluorescence-activated cell sorting (FACS) analysis

To detect ROS, Ki67, and Annexin V, Sertoli cells were collected. The mouse testes were digested with 0.25% trypsin-EDTA to produce a single-cell suspension. Furthermore, the Fixation and Permeabilization Solution Kit (BD, USA) was used to fix and permeabilize the digested cells. The cells were labeled with FITC-conjugated anti-Annexin V (BD, USA), PE-conjugated anti-Ki67 (BD, USA), and PE-conjugated anti-ROS (Abcam, USA) antibodies and their isotype controls at 4 °C for 30 min. After that, flow cytometry (Beckman, USA) was used for analysis according to the manufacturer’s instructions.

### BrdU assay and immunofluorescence of SCP3, SIRT1, PPLZF, P62, and LC3AB

First, the slides were dewaxed with xylene, rehydrated with isopropyl alcohol, incubated with a proteinase K solution, fixed with formaldehyde, and eventually incubated in a DNA labeling solution and an antibody solution. Anti-scp3 antibody, anti-SIRT1 antibody, anti-PLZF antibody, anti-P62 antibody, and anti-LC3AB antibody (Abcam, USA) were used for backstaining, and 4% PFA was used for fixing (Sigma, USA); after that, 0.1% Triton X-100 (Sigma, USA) was used for permeating, and 4% bovine serum albumin (BSA, Sigma, USA) was used for blocking. The slides were incubated with the above five antibodies, and BrdU staining was performed overnight at 4 °C. The sections were stained with FITC-conjugated secondary antibodies (Jackson ImmunoResearch, West Grove), stained with Hoechst 33342 (Beyotime Biotechnology, China), and evaluated with a fluorescence microscope (Olympus, Japan).

### Statistical analysis

All the experiments in this study were repeated at least three times. Data are expressed as the mean ± standard deviation. SPSS 22.0 software was used for one-way ANOVA to analyze significant differences, which were defined as a *P* value less than 0.05.

## Results

### hPMSCs restored disrupted spermatogenesis and raised the levels of testosterone in a mouse model of busulfan-induced testicular toxicity

**Fig. 1:**
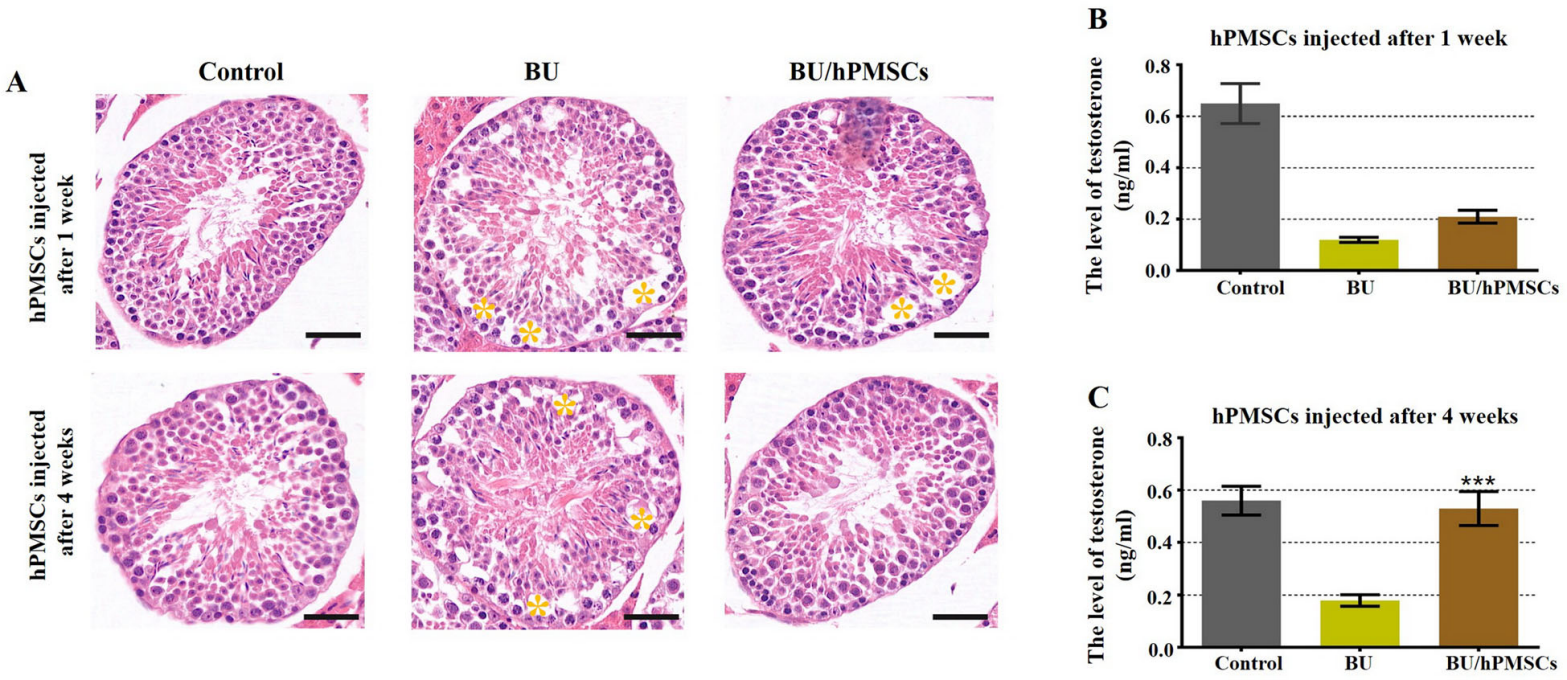
Human placental mesenchymal stem cells (hPMSCs) rescued spermatogenesis and promoted testosterone levels in a busulfan-induced testis toxicity mouse model. **a** Images of HE-stained mouse testis sections in the three groups (the control group and the 1- and 4-week hPMSC treatment groups). Scale bar = 20 μm. *n* = 3 for each group. **b** Testosterone levels were examined 1 week after hPMSC injection in the three groups. **c** Testosterone levels were detected 4 weeks after hPMSC treatment in the three groups. The error bars indicate the SD. ***p < 0.001

To investigate the therapeutic potential of hPMSCs to restore busulfan-disrupted spermatogenesis, testosterone levels and phenotypic characteristics of the vas deferens in the three groups were determined by ELISA and HE staining, respectively (Fig. 1). The histopathological pictures show that busulfan markedly damaged spermatogenic cells, while complete spermatogenesis occurred in the control group. After the busulfan treatment, obvious vacuolation (marked by a yellow quincunx) was observed in the basal compartment in paraffin sections (Fig. 1a). The results suggested that the testis toxicity mouse model induced by busulfan was successfully established. One week after hPMSC injection, cell vacuolation in the basement membrane was slightly reduced. Surprisingly, cell vacuolation disappeared entirely in the hPMSC-treated group at 4 weeks. Compared with that in the BU group, the testosterone level in the HPMSC group was slightly increased at 1 week (Fig. 1b). Nevertheless, the testosterone level of the hPMSC-treated group was greatly increased at 4 weeks compared with that of the BU group (Fig. 1c).

### hPMSCs promoted improved semen parameters and increased weight and size of testes in a mouse model of busulfan-induced testicular toxicity

**Fig. 2:**
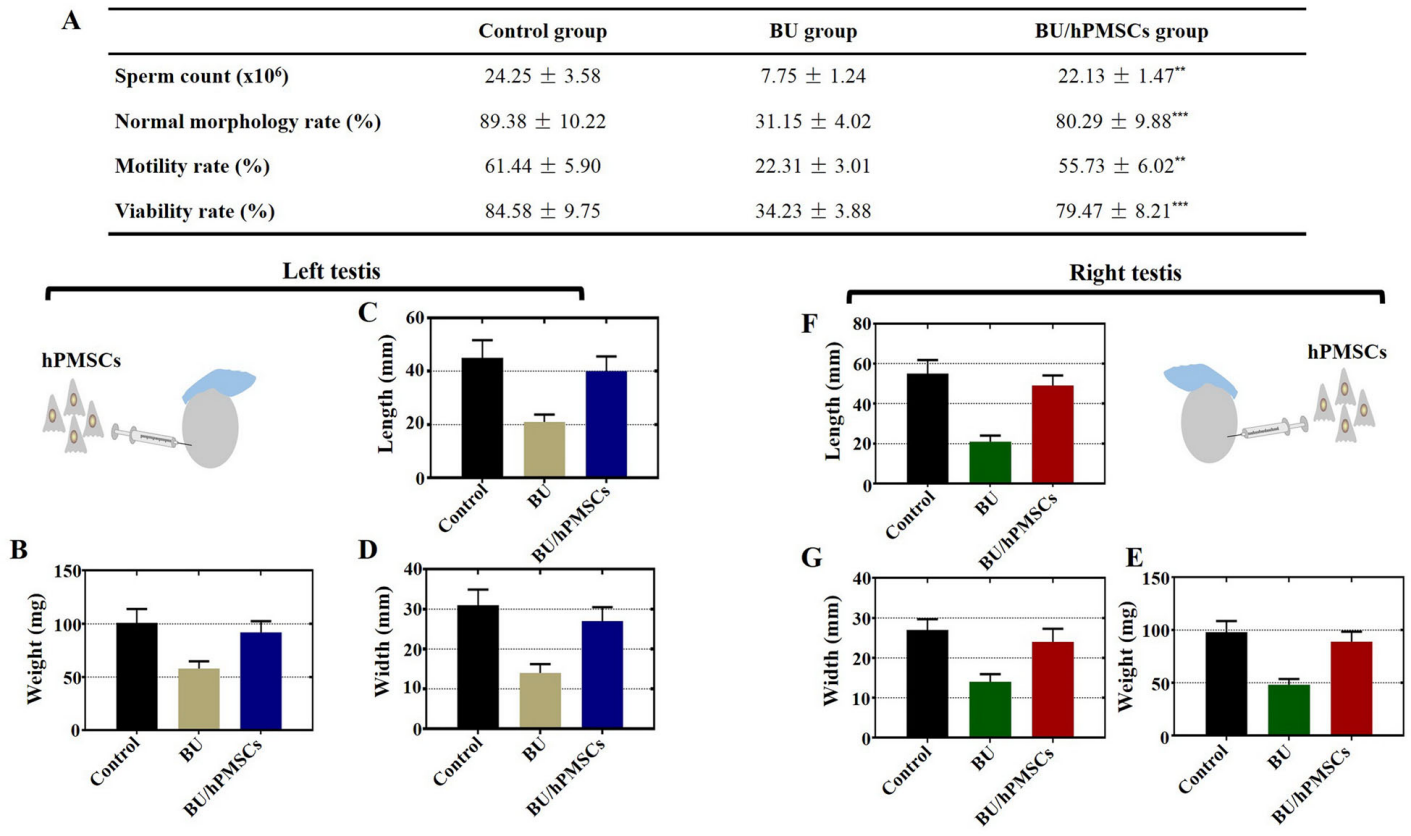
hPMSCs promoted increased testicular weight and size and improved semen parameters in a busulfan-induced testis toxicity mouse model. **a** The sperm count, normal morphology, motility, and proportion of viable cells were determined in the three groups after hPMSC therapy. **b**–**d** Variation in the weight, length, and width of the mouse left testis in the three groups after hPMSC treatment. **e**–**g** Variation in the weight, length, and width of the mouse right testis in the three groups after hPMSC treatment. The error bars indicate the SD. **p < 0.01,***p < 0.001

The sperm count of the BU group presented a more than threefold decline compared to that of the control group. The sperm count returned to a level similar to that of the control group at 4 weeks after hPMSC therapy (Fig. 2a). Next, the ratio of sperm cells with normal morphology to those with abnormal morphology decreased greatly in the BU group but rapidly recovered in the BU/hPMSC group compared with that in the control group (Fig. 2a). Additionally, we observed a nearly threefold decrease in the proportion of motile cells in the BU group, and this proportion was restored at 4 weeks after hPMSC treatment (Fig. 2a). Similarly, the proportion of viable cells declined dramatically in the BU group but returned to the level of the control group at 4 weeks after hPMSC therapy (Fig. 2a). Finally, to determine whether hPMSCs restore testicular function, we measured and compared the weight, length, and width of the mouse left and right testes among the three groups. In the BU group, the width, length, and weight of both the right and left testes were reduced by half. Moreover, the three parameters above markedly increased to normal levels at 4 weeks after hPMSC treatment (Fig. 2b–g).

### hPMSCs suppressed the apoptosis of Sertoli cells and enhanced the rate of proliferation

**Fig. 3:**
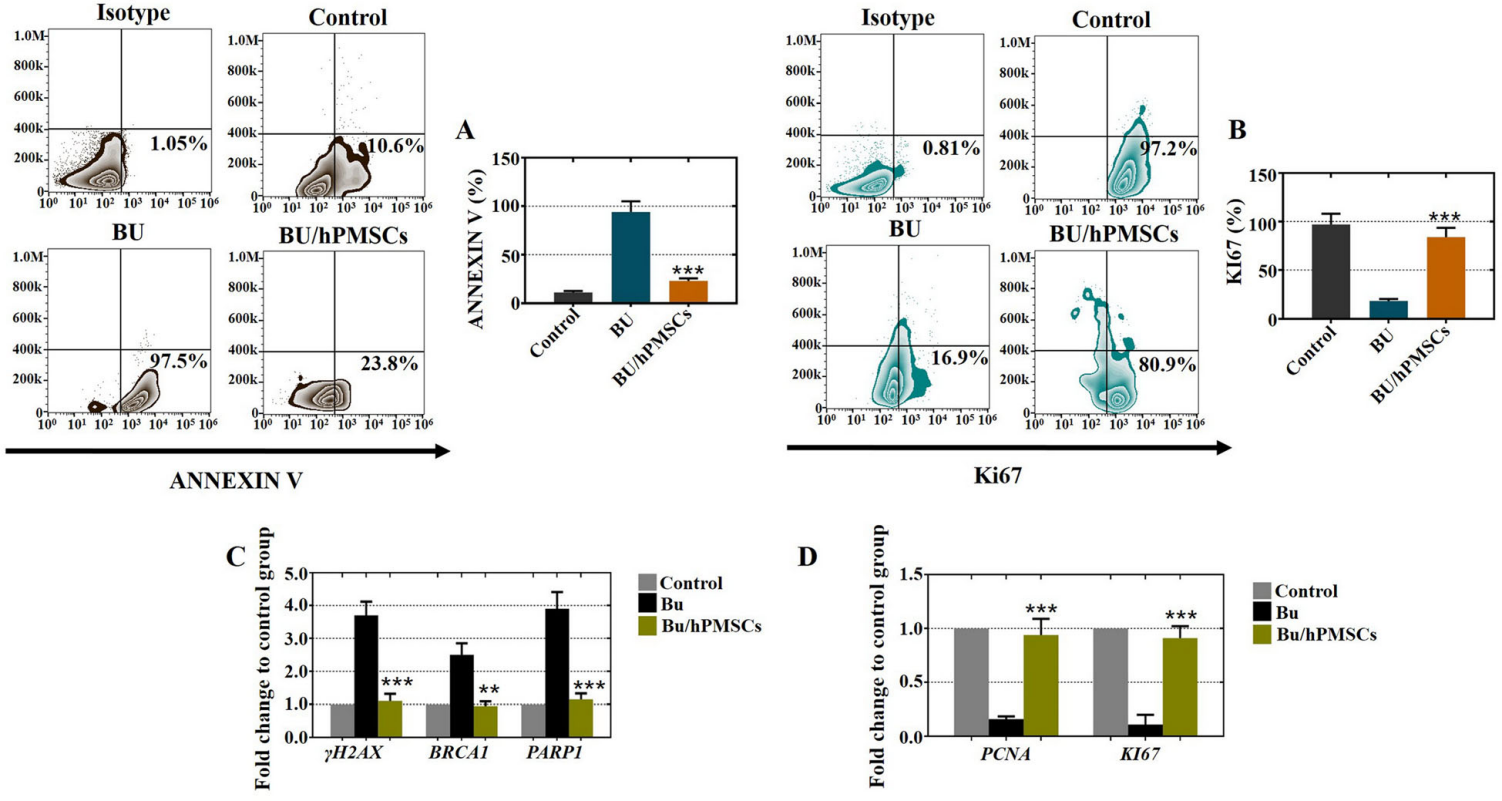
In cultured Sertoli cells treated with busulfan, hPMSCs inhibited cell apoptosis and enhanced cell proliferation. **a** The FACS results indicated that hPMSC treatment inhibited the rate of apoptosis (Annexin V) in Sertoli cells. **b** The FACS results showed that hPMSC treatment improved the proliferation rate (Ki67) of Sertoli cells. **c**, **d** The qRT-PCR results showed that hPMSC treatment suppressed the expression of apoptotic genes (γ-H2AX, BRCA1, and PARP1) and increased the expression of proliferative genes (PCNA and Ki67). All experiments were carried out three times, and the error bars indicate the SD. **p < 0.01, ***p < 0.001

To investigate the role of hPMSCs in enhancing the proliferation and inhibiting the apoptosis of busulfan-induced Sertoli cells, FACS analysis was employed to quantitatively assess cell viability by the proliferation marker of Ki67 and the apoptosis marker of ANNEXIN V. As shown in Fig. 3a, in the BU/hPMSC group, the rate of apoptosis significantly decreased by 23.8%, which was similar to that of the control group but lower than that of the BU group. Additionally, the proliferation rate of the BU/hPMSC group increased by 80.9%, which was higher than that of the BU group (Fig. 3b). Moreover, we used qRT-PCR to detect the expression levels of apoptotic genes (γ-H2AX, BRCA1, and PARP1) and proliferative genes (PCNA and Ki67). As shown in Fig. 3c, after hPMSC treatment, the expression levels of γ-H2AX, BRCA1, and PARP1 in the BU/hPMSC group were similar to those in the control group. The expression patterns of PCNA and Ki67 in the BU/hPMSC group were similar to those in the control group but were greatly decreased in the BU group (Fig. 3d). Briefly, hPMSCs suppressed the apoptosis of Sertoli cells and enhanced the rate of proliferation.

### hPMSCs enhanced cell proliferation and alleviated apoptosis in vitro

**Fig. 4:**
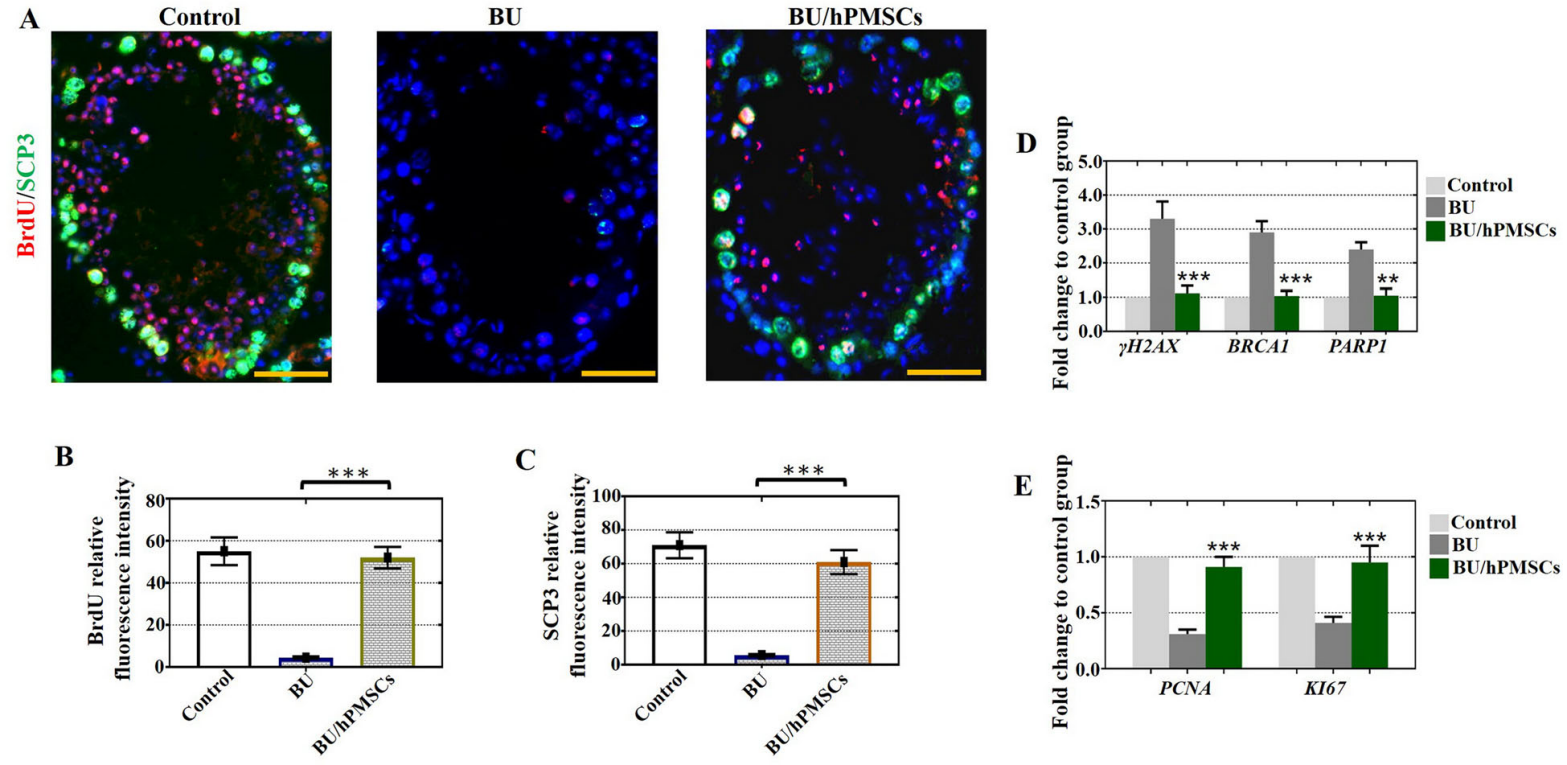
hPMSCs improved cell proliferation and alleviated apoptosis of testes in the busulfan-induced testis toxicity mouse model. **a**–**c** The fluorescence intensities of BrdU (red) and SCP3 (green) in the three groups were detected by fluorescence microscopy. **d**, **e** The mRNA expression of γ-H2AX, BRCA1, PARP1, PCNA, and Ki67 was determined by qRT-PCR. The error bars indicate the SD. **p < 0.01, ***p < 0.001

To explore whether hPMSCs boosted cell proliferation and alleviated apoptosis in vitro, a mouse model of busulfan-induced testicular toxicity was introduced to assess cell apoptosis and proliferation features after hPMSC injection. As expected, hPMSC treatment successfully increased the relative fluorescence intensities of BrdU and SCP3 in the BU/hPMSC group compared with the BU group (Fig. 4a–c). In agreement with the fluorescence microscopy results, we observed a rising trend in the mRNA levels of PCNA and Ki67 in the BU/hPMSC group compared with the BU group (Fig. 4e). Moreover, the mRNA levels of apoptotic genes such as γ-H2AX, BRCA1, and PARP1 revealed a decreasing trend in the BU/hPMSC group compared with the BU group (Fig. 4d).

### hPMSCs increased oxidative protection and decreased oxidative damage in a mouse model of busulfan-induced testicular toxicity

**Fig. 5:**
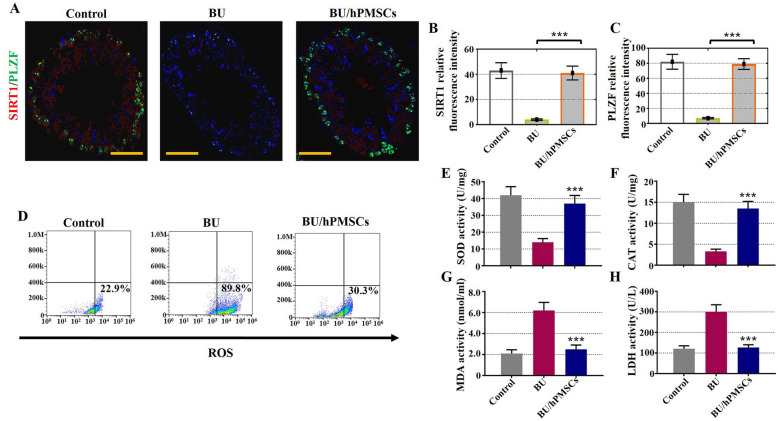
hPMSCs alleviated oxidative damage in a busulfan-induced testis toxicity mouse model. **a**–**c** Fluorescence microscopy was used to detect the relative fluorescence intensity of SIRT1 (red) and PLZF (green) in the three groups. **d** FACS analysis was applied to measure the percentage of ROS^+^ Sertoli cells in the three groups. **e**–**h** ELISA analysis was employed to examine the expression of SOD, CAT, MDA, and LDH. The error bars indicate the SD. ***p < 0.001

We next sought to verify whether hPMSCs protect against oxidative stress and decrease oxidative damage in the busulfan-induced testis toxicity mouse model. The results of fluorescence microscopy, FACS, and ELISA indicated that hPMSCs rescued spermatogenic function by suppressing oxidative stress. As observed by fluorescence microscopy, hPMSCs successfully enhanced the relative fluorescence intensities of SIRT1 and PLZF in the BU/hPMSC group compared with the BU group (Fig. 5a–c). FACS analysis showed that after hPMSC treatment, the proportion of ROS^+^ Sertoli cells was significantly lower in the BU/hPMSC group (30.3%) than in the BU group (89.8%) (Fig. 5d). Finally, the levels of antioxidant enzymes such as SOD and CAT recovered and maintained an increasing trend in the BU/hPMSC group, while MDA and LDH showed a declining trend.

### hPMSCs promoted autophagy in a mouse model of busulfan-induced testicular toxicity

**Fig. 6:**
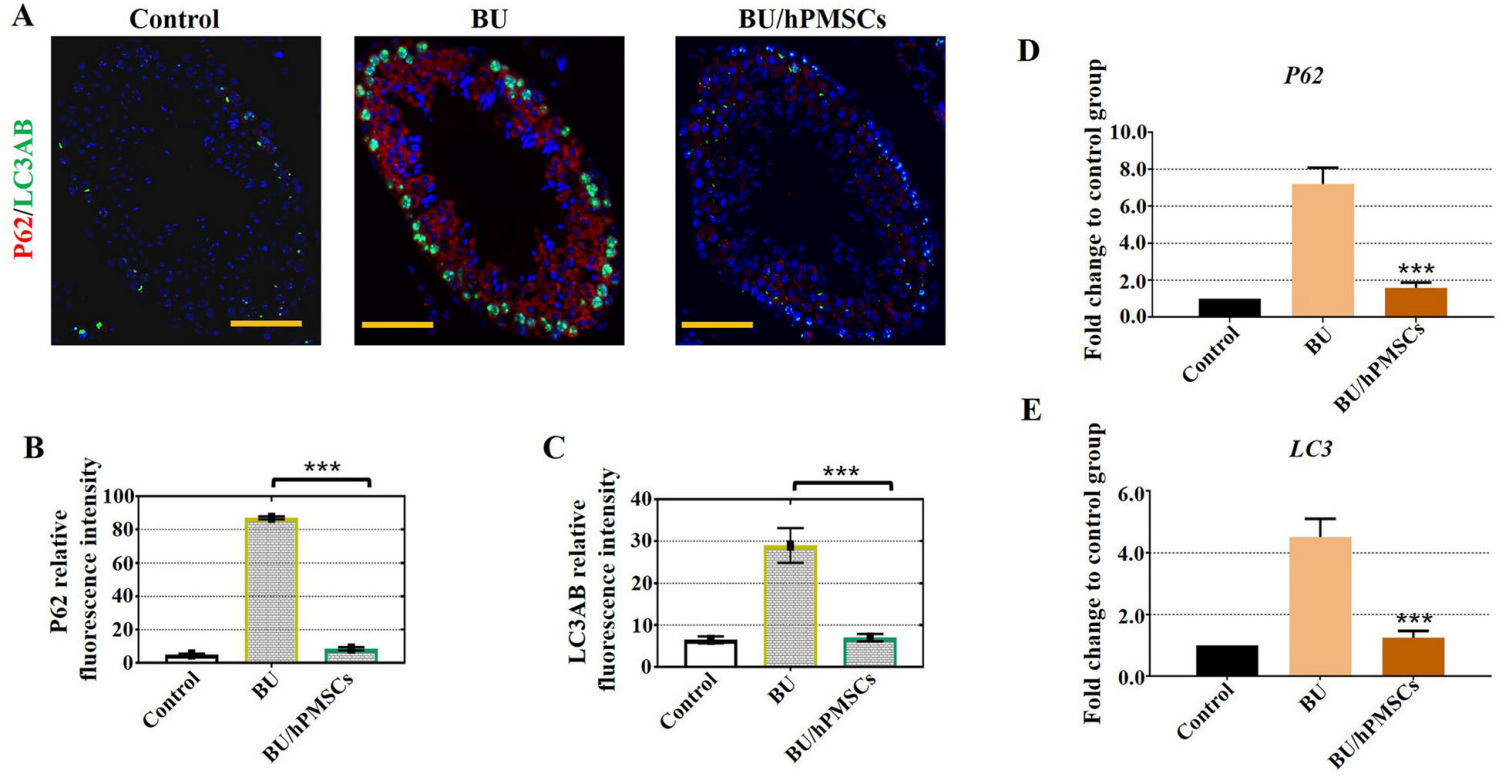
hPMSCs promoted autophagy in a busulfan-induced testis toxicity mouse model. **a**–**c** Fluorescence microscopy was employed to monitor the relative fluorescence intensities of p62 (red) and LC3AB (green) in the three groups. **d**–**e** qRT-PCR assays revealed the mRNA levels of the autophagy-related genes P62 and LC3. The error bars indicate the SD. ***p < 0.001

To further confirm the relationship between autophagy and spermatogenesis, fluorescence microscopy was used to investigate the colocalization of autophagy-associated proteins, including p62 and LC3. The results showed that the immunofluorescence signals of the autophagy markers p62 and LC3 increased in the BU group (Fig. 6a, b), indicating that autophagy was inhibited under busulfan induction. However, LC3 fluorescence vanished at 4 weeks after hPMSC treatment (Fig. 6a, b). A hallmark of autophagy, LC3 acts as a scaffold to recognize p62 and regulate protein turnover in autophagy. Moreover, the fluorescence of p62, a specific substrate of autophagy and an LC3-binding protein, disappeared at 4 weeks after hPMSC treatment. Additionally, in agreement with the immunofluorescence results, the mRNA levels of p62 and LC3 showed trends similar to those of their protein levels (Fig. 6d–e).

## Discussion

Chemotherapy with busulfan is an effective treatment for leukemia, especially in children. Nevertheless, the male reproductive system could be damaged by busulfan and develop oligospermia or azoospermia and finally permanent male sterility [24, 25]. In addition, previous studies have reported that male sterility in mice induced by busulfan is similar to that in humans [8, 26]. In the current research, we found that hPMSC treatment restored spermatogenesis in a mouse model of busulfan-induced testicular toxicity and boosted the proliferation of busulfan-induced mouse Sertoli cells by reducing apoptosis/oxidative stress and promoting autophagy. Consequently, we sought to determine the underlying mechanisms by which hPMSCs improve spermatogenesis by using different analyses.

The previous studies have shown that transplantation of testicular endothelial cells alone could restore spermatogenesis in mice after chemotherapy-induced depletion of spermatogonial stem cells (SSC) [27], autologous spermatogonial stem cell (SSC) transplantation could rescue some forms of male infertility caused by Cldn11 deficiency [28] or alginate oligosaccharides improved testis and blood metabolomes to support the recovery of spermatogenesis [29]. Our research also confirmed that hPMSCs are ideal for restoring spermatogenesis in the testes of mice induced by busulfan [22]. However, the interaction between transplantation of hPMSCs and testis toxicity induced by busulfan has not been explored. By using histological analysis, we confirmed that the number of seminiferous tubules with fluid-filled cavities decreased obviously after hPMSC transplantation (Fig. 1). Additionally, the testosterone level of the hPMSC-treated group returned to normal at 4 weeks after hPMSC treatment, which agreed with the results of our preceding study [22] (Fig. 1). The weight, size, and semen parameters of the testis recovered to normal values after hPMSC injection (Fig. 2). In accordance with our outcomes, busulfan-induced testes of mice transplanted with TECs were similar in size to those of control-injected mice [27]. Our results revealed that the overall number of sperm cells as well as the ratio of sperm cells with normal morphology to those with abnormal morphology were obviously elevated after hPMSC injection (Fig. 2).

Busulfan treatment was shown to lead to ROS-mediated apoptosis [30]; apoptosis is a crucial process in testis development because it manages the proportion of germ cells and Sertoli cells to maintain efficient spermatogenesis [31]. Our study showed that hPMSCs have the ability to suppress the apoptosis of Sertoli cells and enhance their rate of proliferation in vivo and in vitro (Figs. 3 and 4). Moreover, our previous research also confirmed this conclusion since it demonstrated that hPMSCs alleviated cell apoptosis and enhanced cell proliferation to improve premature ovarian insufficiency [12]. ROS are inhibited in stem cell self-renewal and believed to be destructive for spermatogenesis [7]. Our results demonstrated that treatment with hPMSCs suppressed the levels of ROS (Fig. 5). Similar to our findings, hPMSCs were shown to protect against oxidative damage in CD4^+^ T cells by activating Akt-regulated Nrf2 antioxidant signaling [32].

Early studies discovered that the lysosomes of the seminiferous epithelium displayed a cyclical pattern and that autophagy was active in Sertoli cells [33, 34]. In the current study, we found that the autophagy markers p62 and LC3 aggregated in Sertoli cells and mouse testes after busulfan induction not only at the protein level but also at the mRNA level (Fig. 6). As two primary markers of autophagy, p62 and LC3 accumulate when autophagy is inhibited and are reduced when autophagy is induced [35]. Additionally, autophagy is inhibited by mTOR after busulfan treatment in mouse spermatogonial progenitor cells [36]. Collectively, the results from these studies showed that autophagy protected spermatogenesis and Sertoli cells from busulfan-induced stress, which may provide evidence for an important role for hPMSCs in the clinic.

## Conclusions

In this study, we investigated the ability of hPMSCs to protect spermatogenic cells against busulfan-induced injury. By upregulating proliferation markers (Ki67, PCNA, BrdU, and SCP3), antioxidant markers (SIRT1, PLZF, SOD, and CAT), and autophagy markers (p62 and LC3AB) and downregulating apoptosis markers (Annexin V, γ-H2AX, BRCA1, and PARP1) and oxidative markers (MDA and LDH), hPMSCs have substantial potential for repairing busulfan-disrupted spermatogenesis through repressing oxidative stress and apoptosis and enhancing cell proliferation and autophagy. Therefore, these results implied that hPMSCs played a vital role in enhancing cell proliferation and autophagy as well as inhibiting apoptosis and oxidative stress to restore male fertility impaired by busulfan.

## Supplementary Information

**Additional file 1: Supplemental Table 1.** Designations, sequences, and the sizes of real-time PCR amplicons.

## Data Availability

All the data generated or analyzed during this study are included in this published rticle.

## Abbreviations

hPMSCs: Human placental mesenchymal stem cells
ROS: Reactive oxygen species
MSCs: Mesenchymal stem cells
SSC: Spermatogonial stem cell
SPCs: Spermatogonial progenitor cells
ELISA: Enzyme-linked immunosorbent assay
FACS: Fluorescence-activated cell sorting
RT-PCR: reverse transcription-polymerase chain reaction
qPCR: Quantitative polymerase chain reaction
HE: Hematoxylin and eosin
MDA: Malondialdehyde
SOD: Superoxide dismutase
CAT: Catalase
LDH: Lactate dehydrogenase
