# Supplementary material for: Human placental mesenchymal stem cells ameliorate chemotherapy-induced damage in the testis by reducing apoptosis/oxidative stress and promoting autophagy
Source: Stem Cell Res Ther. 2021 Mar 20;12:199. doi: 10.1186/s13287-021-02275-z (PMC7981860; doi:10.1186/s13287-021-02275-z)
Supplement: Supplementary file 1 — Additional file 1: Supplemental Table 1. Designations, sequences, and the sizes of real-time PCR amplicons. [file 13287_2021_2275_MOESM1_ESM.doc]

**Supplemental Table 1 Designations, sequences, and the sizes of real-time PCR amplicons**

**H=Human; M=Mouse.**

| **Name** | **Sequence from 5'-3'** | **Size (bp)** |
| --- | --- | --- |
| BRCA1 (H) Fw | AATGCTGATCCCCTGTGTGA | 70 |
| BRCA1 (H) Rev | CTCTAGGATTCTCTGAGCATGGC |
| PCNA (H) Fw | CTGAGAGCGGAGGACAATGC | 119 |
| PCNA (H) Rev | GGAATTCCAAGCTGCTCCAC |
| PARP1 (H) Fw | AACTGTATTCAGCGGCGACA | 82 |
| PARP1 (H) Rev | ACTCTTCAGTGTGCGGAAGG |
| γH2AX (H) Fw | TTGATTGCCGGGCTTAGAGG | 223 |
| γH2AX (H) Rev | CTGCGGCAGGTATAGAACTC |
| KI67 (H) Fw | GCTTGTTTGGAAGGAGGAAATGT | 94 |
| KI67 (H) Rev | GCCGTCTTAAGGGAGGGCT |
| P62 (H) Fw | GGAAAGGGCTCAATGAGAGACA | 124 |
| P62 (H) Rev | CCTCACATGGGGGTCCAAAG |
| LC3 (H) Fw | GCGAGTTACCTCCCGCAG | 210 |
| LC3 (H) Rev | GTACCTCCTTACAGCGGTCG |
| GAPDH (H) Fw | GAAGGTCGGAGTCAACGGATTT | 223 |
| GAPDH (H) Rev | CTGGAAGATGGTGATGGGATTTC |
| BRCA1 (M) Fw | ATGGGCATGTCGTGAGTTGT | 194 |
| BRCA1 (M) Rev | TCTGGGTGTTGTTTGGTGCT |
| PCNA (M) Fw | AAAGATGCCGTCGGGTGAAT | 179 |
| PCNA (M) Rev | TGGTTACCGCCTCCTCTTCT |
| PARP1 (M) Fw | GACGCCGAGGCGATTCAAA | 100 |
| PARP1 (M) Fw | CAGCCATCCTCTCGTCCAGT |
| γH2AX (M) Fw | TTGATTGCCGGGCTTAGAGG | 223 |
| γH2AX (M) Fw | CTGCGGCAGGTATAGAACTC |
| KI67 (M) Fw | ACCATCATTGACCGCTCCTTT | 209 |
| KI67 (M) Fw | AGGCCCTTGGCATACACAAA |
| GAPDH (M) Fw | TTCCAGTATGACTCTACCCACGGCA | 137 |
| GAPDH (M) Rev | GCACCAGCATCACCCCATTTG |
